# Prognostic value of tumor–stroma ratio combined with the immune status of tumors in invasive breast carcinoma

**DOI:** 10.1007/s10549-017-4617-6

**Published:** 2017-12-22

**Authors:** K. M. H. Vangangelt, G. W. van Pelt, C. C. Engels, H. Putter, G. J. Liefers, V. T. H. B. M. Smit, R. A. E. M. Tollenaar, P. J. K. Kuppen, W. E. Mesker

**Affiliations:** 1 Department of Surgery, Leiden University Medical Center, Albinusdreef 2, 2333 ZA Leiden, The Netherlands; 2 Department of Pathology, Leiden University Medical Center, Leiden, The Netherlands; 3 Department of Medical Statistics, Leiden University Medical Center, Leiden, The Netherlands

**Keywords:** Breast cancer, Tumor–stroma ratio, Immune cells, HLA, Prognosis

## Abstract

**Purpose:**

Complex interactions occur between cancer cells and cells in the tumor microenvironment. In this study, the prognostic value of the interplay between tumor–stroma ratio (TSR) and the immune status of tumors in breast cancer patients was evaluated.

**Methods:**

A cohort of 574 breast cancer patients was analyzed. The percentage of tumor stroma was visually estimated on Hematoxylin and Eosin (H&E) stained histological tumor tissue sections. Immunohistochemical staining was performed for classical human leukocyte antigen (HLA) class I, HLA-E, HLA-G, markers for regulatory T (Treg) cells, natural killer (NK) cells and cytotoxic T-lymphocytes (CTLs).

**Results:**

TSR (*P* < .001) and immune status of tumors (*P* < .001) were both statistically significant for recurrence free period (RFP) and both independent prognosticators (*P* < .001) in which tumors with a high stromal content behave more aggressively as well as tumors with a low immune status. Ten years RFP for patients with a stroma-low tumor and high immune status profile was 87% compared to 17% of patients with a stroma-high tumor combined with low immune status profile (*P* < .001). Classical HLA class I is the most prominent immune marker in the immune status profiles.

**Conclusions:**

Determination of TSR is a simple, fast and cheap method. The effect on RFP of TSR when combined with immune status of tumors or expression of classical HLA class I is even stronger. Both are promising for further prediction and achievement of tailored treatment for breast cancer patients.

**Electronic supplementary material:**

The online version of this article (10.1007/s10549-017-4617-6) contains supplementary material, which is available to authorized users.

## Introduction

Survival for patients with invasive breast cancer has increased in the last decade due to new and improved therapeutic options as well as new insights in molecular biology. Methods to select patients based on the tumor phenotype are important to reduce over- and undertreatment, for example, gene expression profiles that identify subtypes [1, 2] associated with higher risk of metastasis. Although these techniques result in prognostic and predictive valuable information for specific patient groups, optimization of risk assessment might benefit from further improvement.

Despite an important update on the role of the microenvironment on cancer development by Hanahan et al. [3, 4], the classification system for predicting metastasis and disease-specific survival is still based on traditional tumor staging criteria (AJCC/UICC-TNM Classification) [5–7] which focus largely on the tumor cell autonomous processes and not on the microenvironment.

Complex interactions occur between cancer cells and cells in the tumor microenvironment, such as immune and stromal cells. A high stromal content has been associated with worse prognosis in different solid cancer types including breast cancer and especially in triple negative breast cancer [8–14]. Together with the development of malignant tumor stroma, the connective tissue framework of the tumor becomes active. The collagen bundles degrade, the number of inflammatory cells increases, fibroblasts differentiate into myofibroblasts and proliferate and angiogenesis increases [15]. Also, the cellular immune response has a fundamental role in cancer development. An example of the prognostic value of the activity of the immune system is represented by the Immunoscore which analyzes the distribution of CD3^+^ lymphocytes and CD8^+^ cytotoxic T cells [16]. In breast cancer, especially in triple negative tumors, the increased presence of tumor-infiltrating lymphocytes has been associated with good prognosis [17, 18]. De Kruijf et al. showed that the immune status of tumors based on six cellular immune markers has a statistically significant effect on prognosis preferable for tumors with a high immune status [19]. These six cellular immune markers (HLA-E, HLA-G, classical HLA class I (HLA-A, HLA-B and HLA-C), natural killer (NK) cells, cytotoxic T-lymphocytes (CTLs) and regulatory T (Treg) cells) were selected based on biological rationale and the balance between their various interactions.

Suggestions have been made about the influence of tumor stroma on suppression of the immune response [9, 20–23]. In this present study, the prognostic value of the interplay between tumor–stroma ratio (TSR) and the immune status of tumors in breast cancer patients was evaluated. We hypothesize that stroma-high tumors in combination with a low immune status behave more aggressively resulting in a high risk of disease progression.

## Materials and methods

### Study population

The study population was assessed retrospectively and consists of primary non-metastasized breast cancer patients. The patients were primarily treated with surgery between 1985 and 1994 in Leiden University Medical Center (*N* = 584). Exclusion criteria were bilateral breast tumors and a history of cancer (other than basal cell carcinoma or cervical carcinoma in situ). The resected breast tumors were graded by experienced breast cancer pathologists using current pathological standards. All samples were handled in a coded fashion, according to national ethical guidelines (“Code for Proper Secondary Use of Human Tissue”, Dutch Federation of Medical Scientific Societies). Approval of the study was obtained from the LUMC Medical Ethics Committee. The recommendations for reporting on tumor markers (the REMARK criteria) in prognostic studies were respected [24].

### Tumor–stroma ratio

The TSR was visually estimated on routine Hematoxylin and Eosin (H&E) stained slides from formalin-fixed paraffin-embedded (FFPE) blocks of the primary tumor (*N* = 584) as previously described by our group [25]. Thirty-two percent of the tissues were scored in a blinded fashion by a second observer, with a concordance of classification of 94% (Cohen’s kappa = .85). Ten tissues were not eligible for TSR scoring due to poor quality. Evaluation of TSR started with microscopical orientation using a 5 × objective. Subsequently, a 10 × objective was used in the most stroma-abundant area. The field of highest stromal percentage was selected and scored per tenfold increments. Tumor cells must be present on all sides (north, east, south and west). Stroma percentage ≤ 50% was categorized as stroma-low and stroma percentage > 50% as stroma-high (Supplementary Fig. 1) [8, 12].

### Immunohistochemistry

Tissue sections from intra-operatively derived FFPE tissue micro-array (TMA) material and immunohistochemistry analysis were used as previously described [19, 26, 27]. Whole FFPE sections were immunohistochemically stained with mouse antibodies against CD8^+^ and PEN5 recognizing CTLs and NK cells, respectively. TMA tissue sections were used for immunohistochemical stainings for the expression of classical HLA class I (anti-HLA-A and anti-HLAB/C), non-classical HLA-E, HLA-G and Treg cell infiltration as previously described in the literature [26, 27].

Quantification of CD8^+^ cells and PEN5 cells was performed in a blinded setup by two independent observers. Tumor infiltration of CD8^+^ was divided into low CTL infiltration (0–100 CD8^+^ tumor infiltrating cells/mm^2^) and high CTL infiltration (100–3.000 CD8^+^ tumor infiltrating cells/mm^2^). Tumor infiltration of NK cells was divided into the presence or absence of NK cells. Classical HLA class I was categorized into loss versus expression and HLA-E divided into no expression versus expression. HLA-G and Treg infiltration were categorized in absent versus present (Supplementary Fig. 2).

**Fig. 1:**
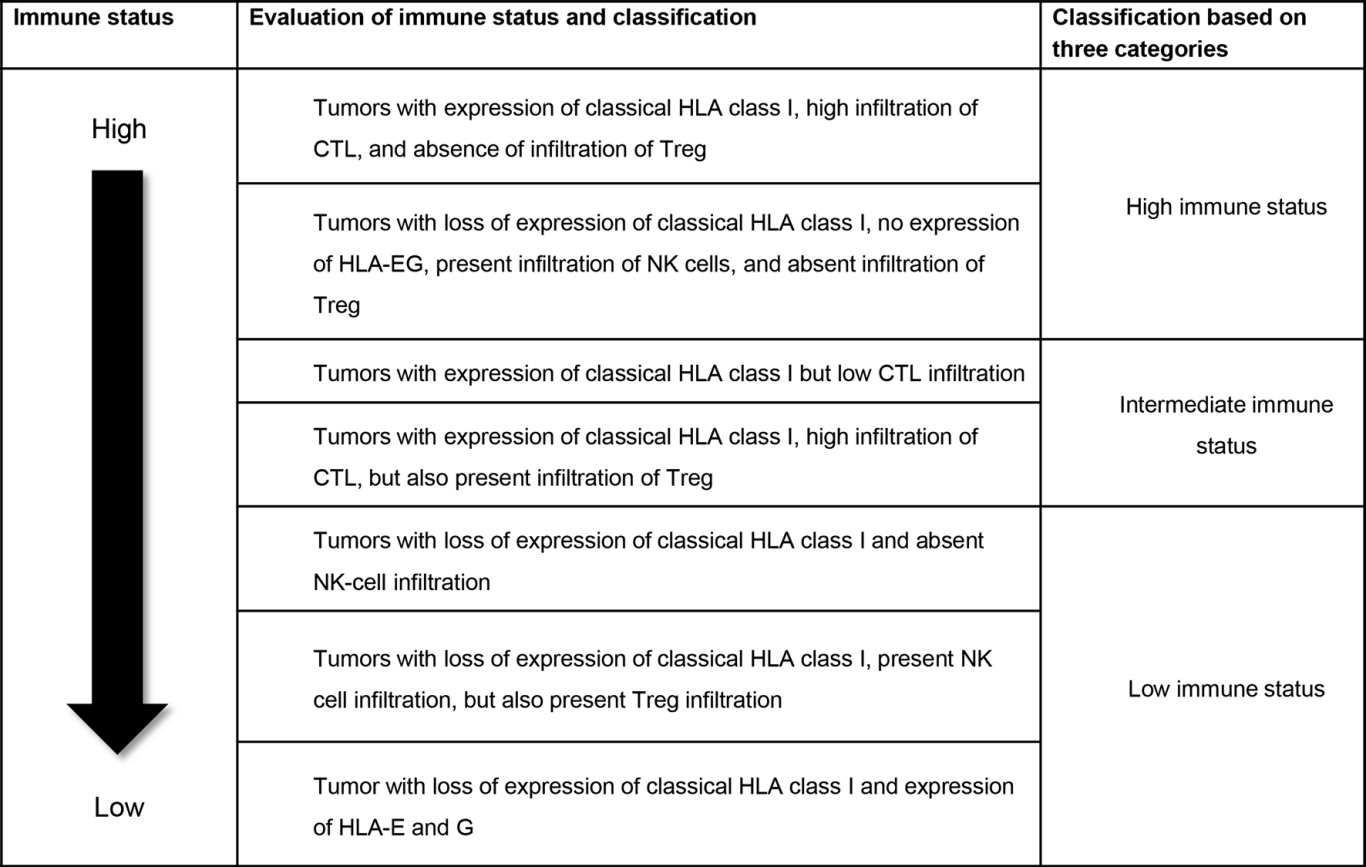
Evaluation of immune status and classification. *HLA* human leukocyte antigen, *CTL* cytotoxic T-lymphocytes, *Treg* regulatory T cells, *NK* natural killer

These six immune markers were classified into three immune status profile groups (Fig. 1) as previously described by de Kruijf et al. for this cohort [19].

### Statistical analysis

Statistical analyses were performed using IBM SPSS statistics (version 23.0 for Windows). The inter-observer agreement in TSR, CTL and PEN5 evaluation is represented by Cohen’s Kappa value. A value above 0.6 was valid. Pearson *χ* ^2^ test was used for the evaluation of statistically significant differences between included and excluded patients, distribution of the separate immune markers between stroma-high and stroma-low cases and three immune status categories. A *P* value < .05 was considered statistically significant. The Kaplan–Meier method was performed to analyze the overall survival (OS) and recurrence free period (RFP). The log-rank test was applied for comparison between these curves. A *P* value < .05 was considered statistically significant. The time from date of surgery until any recurrence of breast cancer was defined as RFP. OS was defined as the time from date of surgery until death from any cause. Univariate and multivariate analyses for RFP and OS were calculated by Cox proportional hazard analysis. Variables with *P* value < .10 in univariate analysis were entered in multivariate analysis. Effect modification was evaluated by adding interaction in Cox regression analysis. Stepwise regression analysis (backward and forward) of the different immune cells was evaluated. Missing values were not included.

## Results

### Patients

**Fig. 2:**
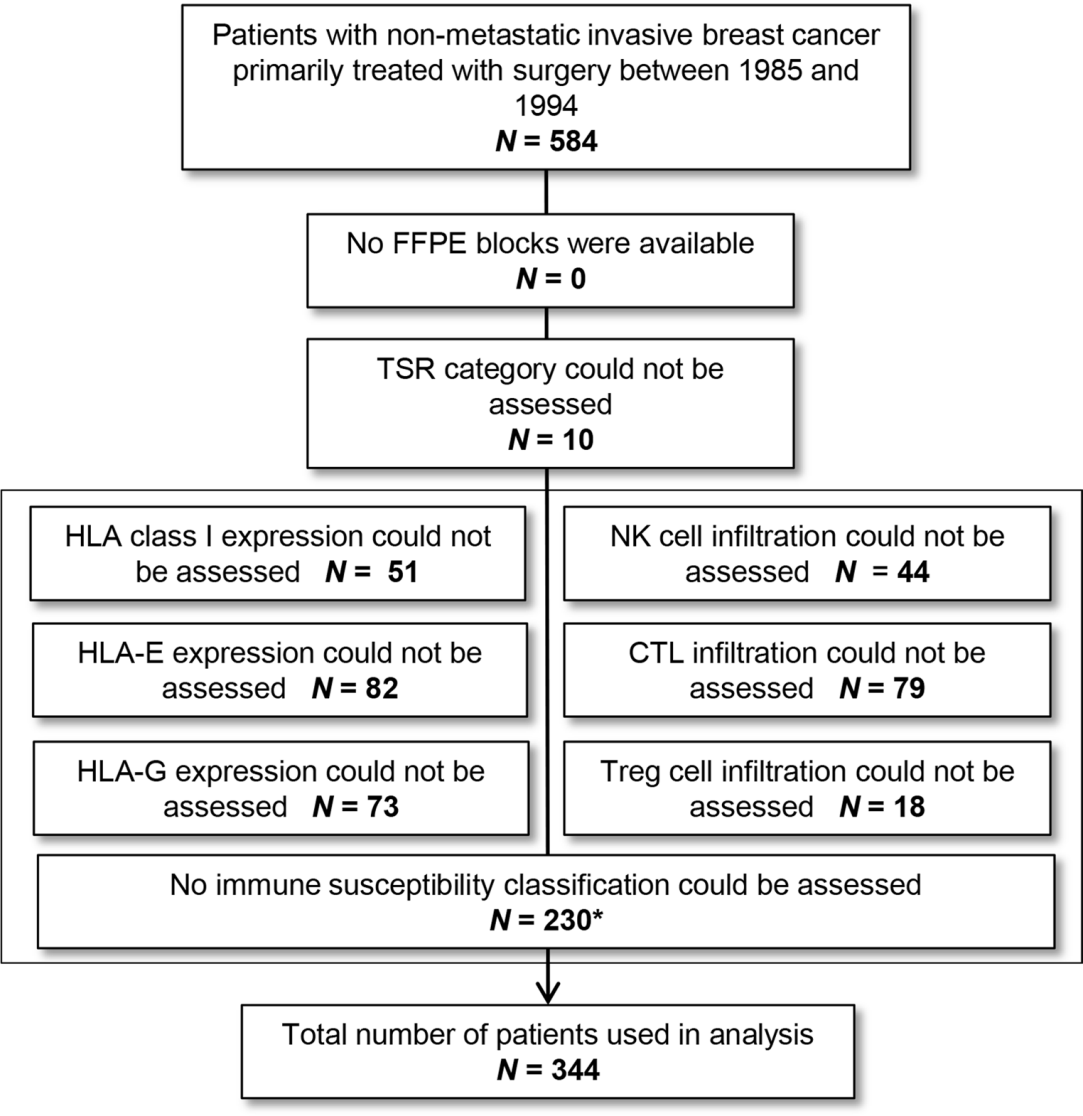
Flowchart of subject inclusion. * For categorizing in one of the three immune status categories not all six groups need to be known. *FFPE* formalin-fixed paraffin-embedded, *NK* natural killer, *CTL* cytotoxic T-lymphocyte, *Treg* regulatory T, *TSR* tumor–stroma ratio

Of all patients (*N* = 584), FFPE blocks were available. TSR could be evaluated in 98% of the cases (*N* = 574). In 43% of the cases, no classification of the immune status could be made due to the low quality of tissues or TMAs. The loss or damage of TMA cores is a known problem associated with preparation, staining and mounting of TMA slides. Moreover, the cores we used were rather small. Since several markers were combined in the profiles, the patient was excluded from further analyses when data of one or more markers were missing. Figure 2 provides a flowchart of subjects included. By comparison of prognostic parameters, no differences were found between included (*N* = 344) and excluded cases (*N* = 230), except for the treatment with hormonal therapy (*P* < .001). This can be explained by the fact that this therapy was only given sporadically between 1985 and 1988. No statistically significant differences were found for age, grade, tumor stage, tumor type, nodal stage, histological type, estrogen receptor, progesterone receptor, HER2 expression, TSR, chemotherapy and radiotherapy in these two groups.

The median follow-up of the 344 included patients was 10.2 years (0.2–22.4 years). The mean age at presentation was 58.0 years (27.5–90.2 years). There is no statistically significant difference in the distribution of the separate markers between stroma-high and stroma-low cases, nor in the three immune status categories (*P* = .30). Table 1 provides a detailed overview of the immune markers stratified by TSR and Table 2 shows the clinicopathological and treatment characteristics.

**Table 1:** Distribution of the separate elements of the three immune status profiles

Characteristics	Stroma-low (*N* = 177)		Stroma-high (*N* = 167)		*P* value
	*N*	%	*N*	%	
HLA class I					.24
Loss or downregulation	98	55.4	103	61.7	
Expression	79	44.6	64	38.3	
HLA-E					.87
Negative	97	54.8	93	55.7	
Positive	80	45.2	74	44.3	
HLA-G					.72
Negative	108	61.0	105	62.9	
Positive	69	39.0	62	37.1	
NK cells					.47
Negative	78	44.1	79	47.3	
Positive	95	53.7	82	49.1	
Missing	4	2.2	6	3.6	
CTL					.19
Low infiltration	115	65.0	121	72.5	
High infiltration	55	31.0	42	25.1	
Missing	7	4.0	4	2.4	
Treg cells					.62
Absence	97	54.8	98	58.7	
Presence	74	41.8	67	40.1	
Missing	6	3.4	2	1.2	
Immune status profiles					.30
High IS	39	22.0	26	15.5	
Intermediate IS	108	61.0	109	65.3	
Low IS	30	17.0	32	19.2	

The subtypes were constructed according to the criteria shown in this table. Only the cases for which both stromal content and immune subtyping could be performed were included in the analyses. HLA Human leukocyte antigen, NK natural killer, CTL cytotoxic T-lymphocyte, Treg regulatory T, IS immune status

**Table 2:** Patient characteristics

	(*N* = 344)	%
Age (in years)		
<40	27	7.9
>40–60	168	48.8
>60	149	43.3
Grade		
I	52	15.1
II	171	49.7
III	118	34.3
Missing	3	0.9
Histological type		
Ductal	309	89.8
Lobular	32	9.2
Missing	3	0.9
Tumor stage		
pT1	121	35.2
pT2	170	49.4
pT3/4	43	12.5
Missing	10	2.9
Nodal stage		
Negative	189	55.0
Positive	147	42.7
Missing	8	2.3
ER status		
Negative	134	39.0
Positive	206	59.9
Missing	4	1.1
PR status		
Negative	139	40.4
Positive	200	58.1
Missing	5	1.5
HER2 status		
Negative	254	73.8
Positive	25	7.3
Missing	65	18.9
Breast cancer subtypes		
Luminal A	192	55.8
Luminal B	10	2.9
HER2-like	15	4.4
Triple-negative	62	18.0
Missing	65	18.9
Surgery and RT		
MST without RT	143	41.6
MST with RT	64	18.6
BCS without RT	1	0.3
BCS with RT	136	39.5
Chemotherapy		
No	265	77.0
Yes	79	23.0
Hormonal therapy		
No	273	79.4
Yes	71	20.6
TSR		
Stroma-low	177	51.5
Stroma-high	167	48.5
Immune status of tumor		
High	65	18.9
Intermediate	217	63.1
Low	62	18.0
Combination TSR and immune status		
Stroma-low/high IS	39	11.3
Stroma-low/intermediate IS	108	31.4
Stroma-low/low IS	30	8.7
Stroma-high/high IS	26	7.6
Stroma-high/intermediate IS	109	31.7
Stroma-high/low IS	32	9.3

*ER* estrogen receptor, *PR* progesterone receptor, *HER2* human epidermal growth factor receptor 2, *MST* mastectomy, *RT* radiotherapy, *BCS* breast conserving therapy, *TSR* tumor–stroma ratio, *IS* immune status

### Prognostic value of the TSR

**Fig. 3:**
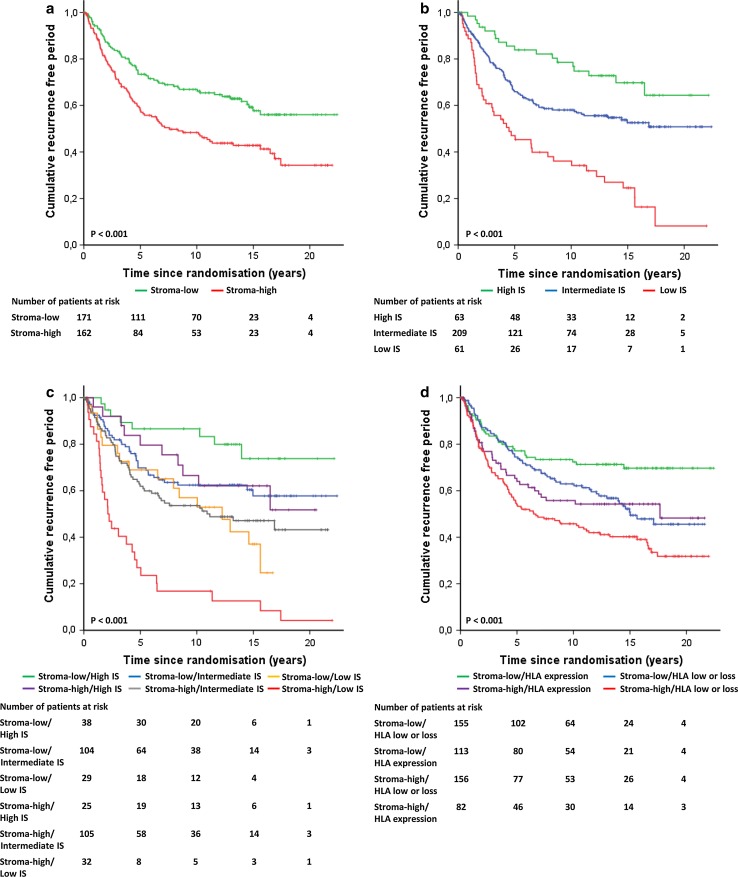
Kaplan–Meier analysis for RFP of TSR, immune status profiles and classical HLA class I. **a** RFP for stroma low and high tumors, **b** RFP for three IS profiles, **c** RFP for TSR combined with IS profiles, **d** RFP for TSR combined with classical HLA class I. *IS* immune status, *RFP* recurrence free period, *TSR* tumor–stroma ratio

Tumors with low and high stromal contents were observed in 51.5 and 48.5% of the cases (*N* = 574), respectively. Patients with stroma-high tumors had a worse RFP (HR 1.75; 95% CI 1.37–2.25; *P* < .001) and OS (HR 1.28; 95% CI 1.04–1.58; *P* = .02) compared to patients with stroma-low tumors (not shown). After 10 years, 32% of the patients with a stroma-low tumor had developed a recurrence of disease compared to 50% of patients with a stroma-high tumor. These results for RFP in favor for stroma-low tumors were also seen in the group of patients (*N* = 344) in which the immune status could be assessed (HR 1.76; 95% CI 1.28–2.42; *P* < .001) (Fig. 3a) with a 10-year RFP of 67% of patients in the stroma-low group compared to 49% in the stroma-high group. OS showed no significant difference between both stroma groups (HR 1.3; 95% CI .095–1.64; *P* = .114). Analysis for breast cancer subgroups showed that patients with a triple negative tumor have a high hazard ratio of 2.4 (95% CI 1.32–4.40; *P* = .003) for RFP in both the total group (known TSR) and in the selected group (known TSR and immune status). Furthermore, within the luminal A subgroup the TSR showed a significant difference in RFP (HR 1.5; 95% CI 1.13–2.19; *P* = .008), but not for OS. For the other subgroups (Luminal B and HER2-like tumors), no prognostic value of the TSR was found (Supplementary Table 1a, b).

### Prognostic value of the immune status of tumors

The immune status of tumors was classified as high in 18.9%, intermediate in 63.1% and low in 18.0% of the breast cancer cases. The RFP (Fig. 3b) and OS curves (not shown) of the three immune status categories were statistically significant (*P* < .001) in which patients with a high immune status profile had a better outcome compared to patients with a low immune status profile. After 10 years of follow-up, 79% of the patients in the high immune status category did not develop recurrence of disease compared to 58% in intermediate immune status category and 36% in low immune status category. Analysis for breast cancer subgroups showed that patients with a luminal A or triple negative tumor have a worse prognosis for both RFP and OS (Supplementary Table 2).

### Prognostic value of TSR and immune status of tumors combined

The RFP data of TSR and immune status subtypes were combined and plotted in Fig. 3c. The overall *P* value between the subgroups was statistically significant (*P* < .001) (Table 3). A trend was observed for stroma-high tumors compared to stroma-low tumors calculated for the high immune status profile (*P* = .15) and intermediate immune status profile (*P* = .08). However, only for the low immune status profile the difference between stroma-high and stroma-low tumors showed significance (*P* = .002). Ten years RFP for stroma-low and high immune status showed a recurrence rate of 87 versus 17% of patients with stroma-high and low immune status tumors.

**Table 3:** Univariate and multivariate analysis for RFP and OS calculated by Cox proportional hazard analysis

Characteristics	*N*	Recurrence free period						Overall survival					
		Univariate analysis			Multivariate analysis			Univariate analyses			Multivariate analysis		
		HR	95% CI	*P* value	HR	95% CI	*P* value	HR	95% CI	*P* value	HR	95% CI	*P* value
Age (in years)													
< 40	27	1		.551				1		<.001			
> 40–60	168	1.08	0.61–1.94					1.62	0.81–3.24				
> 60	149	0.90	0.49–1.64					3.45	1.74–6.83				
Grade													
I	52	1		.004				1		.009			
II	171	1.50	0.89–2.55					1.42	0.91– 2.22				
III	118	2.25	1.32–3.85					1.94	1.23–3.07				
Histological type													
Ductal	309	1		.199				1		.163			
Lobular	32	1.39	0.84–2.30					1.37	0.88–2.14				
Tumor stage													
pT1	121	1		<.001				1		<.001			
pT2	170	1.90	1.31–2.75					1.89	1.36–2.63				
pT3/4	43	2.89	1.76–4.77					3.36	2.19–5.15				
Nodal stage													
Negative	189	1		<.001				1		<.001			
Positive	147	3.02	2.18–4.18					2.06	1.56–2.72				
ER status													
Negative	134	1		.580				1		.264			
Positive	206	0.91	0.66–1.26					0.85	0.65–1.13				
PR status													
Negative	139	1		.290				1		.211			
Positive	200	0.84	0.61–1.16					0.84	0.64–1.11				
HER2 status													
Negative	254	1		.840				1		.174			
Positive	25	1.07	0.58–1.98					1.40	0.86–2.29				
Breast cancer subtypes													
Luminal A	192	1		.823				1		.189			
Luminal B	10	1.06	0.39–2.89					1.17	0.51–2.66				
HER2-like	15	1.15	0.53–2.48					1.74	0.96–3.15				
Triple-negative	62	1.22	0.80–1.84					1.31	0.92–1.87				
Surgery and RT													
MST without RT	143	1		<.001				1		<.001			
MST with RT	64	1.99	1.34–2.95					1.31	0.93–1.85				
BCS without RT	1												
BCS with RT	136	0.76	0.52–1.10					0.47	0.34–0.65				
Chemotherapy													
No	265	1		.976				1		.019			
Yes	79	1.01	0.70–1.45					0.65	0.46–0.93				
Hormonal therapy ER positive													
No	161	1		.242				1		.023			
Yes	45	1.32	0.83–2.10					1.61	1.07–2.43				
Hormonal therapy HER2 positive													
No	17	1		.600				1					
Yes	8	1.39	0.41–4.76					1.71	0.66–4.23	.270			
TSR													
Stroma-low	177	1		<.001	1		<.001	1		.114	1		.048
Stroma-high	167	1.76	1.28–2.42		2.10	1.50–2.93		1.25	0.95–1.64		1.34	1.00–1.80	
Immune status of tumor													
High	65	1		<.001	1		<.001	1		<.001	1		.001
Intermediate	217	1.87	1.13–3.10		2.10	1.22–3.61		1.85	1.22–2.82		1.87	1.19–2.94	
Low	62	4.19	2.43–7.24		4.32	2.40–7.76		2.74	1.70–4.42		2.67	1.60–4.46	
Combination TSR and immune status													
Stroma-low/high IS	39	1		<.001	1		<.001	1		<.001	1		.003
Stroma-low/intermediate IS	108	2.11	0.99–4.51		2.70	1.13–6.46		1.83	1.04–3.20		1.74	0.94–3.23	
Stroma-low/low IS	30	3.53	1.52–8.18		4.75	1.84–12.27		2.00	1.02–3.90		2.39	1.16–4.93	
Stroma-high/high IS	26	1.95	0.77–4.94		2.86	1.03–7.94		1.06	0.49–2.30		1.15	0.50–2.61	
Stroma-high/intermediate IS	109	3.09	1.47–6.49		5.00	2.11–11.85		1.97	1.13–3.45		2.29	1.24–4.23	
Stroma-high/low IS	32	9.25	4.21–20.31		11.54	4.63–28.79		3.97	2.12–7.46		3.27	1.64–6.52	

*ER* estrogen receptor, *PR* progesterone receptor, *HER2* human epidermal growth factor receptor 2, *MST* mastectomy, *RT* radiotherapy, *BCS* breast conserving therapy, *TSR* tumor–stroma ratio, *IS* immune status

Table 3 shows the results of univariate and multivariate Cox regression analyses. TSR remained statistically significant for RFP (*P* < .001) in multivariate Cox regression analysis and the immune status for RFP (*P* < .001) and OS (*P* = .001). Effect modification of stroma and immune status was not statistically significant. As expected, the TSR combined with immune status showed additional prognostic value in the analyzed patient cohort.

### Prognostic value of TSR combined with classical HLA class I

To evaluate whether one or more of the six cellular immune cells were decisive in the immune status categories, a stepwise regression analysis was performed. In this analysis, classical HLA class I showed to be statistically significant in the immune status categories for RFP (*P* = .007), but not for OS (*P* = .06), whereas the other immune cells were not. These results indicate that classical HLA class I is the most determinant factor in the three immune status profiles. In 523 of the 574 cases (91%), classical HLA class I could be assessed. Tumors expressing classical HLA class I had significantly less recurrences (*P* = .001), with 10 years RFP of 66 versus 55%. In the same group, TSR showed RFP of 67 versus 49% in benefit for stroma-low tumors (*P* < .001).

Figure 3d shows a statistically significant difference (*P* < .001) for RFP for the combination of TSR and classical HLA class I. This indicates that patients with a stroma-low tumor and expression of classical HLA class I have a better prognosis compared to patients with a stroma-high tumor and loss of expression or downregulation of classical HLA class I with 10-year RFP 72% versus 46%, respectively.

In triple negative tumors, classical HLA class I (*N* = 92) was also of prognostic value (HR 0.28; 95% CI 0.15–0.55; *P* < .001). Patients with loss of expression or downregulation of classical HLA class I showed a 10-year RFP of 35% compared to 73% of the patients in which HLA class I is expressed. TSR and classical HLA class I combined showed significant difference in RFP (*P* = .001). Patients with stroma-low tumors and expression of classical HLA class I showed fewer recurrences compared to patients with stroma-high tumors and loss of expression or downregulation of classical HLA class with 10-year RFP of 75 versus 26%, respectively.

## Discussion

There is a growing body of evidence that TSR and immune cell response in cancer development might be important factors in patient stratification for treatment decision making. The relation of the stromal involvement and immune response for the determination of patients for adjuvant treatment has merely been investigated. Gujam et al. described the relationship between TSR and clinicopathological parameters as tumor inflammatory infiltrate, CD68^+^ macrophage infiltrate and CD4^+^ and CD8^+^ T-lymphocyte infiltrate in ductal breast cancer. They concluded that a high TSR was consistently associated with low tumor inflammatory infiltrate [9]. Hynes et al. also published on the combination of TSR with peritumoral diffuse lymphoid inflammation and Crohn’s disease-like reaction in stage II/III colon cancer. A combination of these three parameters showed a significant association with survival outcomes [23].

Our study showed that TSR and the combination of six cellular immune cells, categorized into three immune status subgroups, are both independent prognostic factors. A combination of both parameters even strengthens each other’s’ effect.

The six cellular immune cells were selected based on biological rationale and the balance between their various interactions. Classical HLA class I presents tumor-associated antigens on the cell surface. CTLs are capable of recognizing the presentation of these antigens by HLA-A, HLA-B or HLA-C [28]. Tumor cells can escape recognition by CTLs by losing classical HLA class I expression. This makes the tumor cells more prone for recognition by NK cells [29]. On the other hand, HLA-E and HLA-G, also known as non-classical HLA class I, play a crucial role in the immune surveillance by NK cells. Expression of non-classical HLA I has an inhibitory effect on the function of NK cells [29–31]. Other cells which are important in tumor development are Tregs. Tumor cells can escape immune surveillance by attraction and induction of Tregs [32].

In this study, the prognostic value of TSR in addition with classical HLA class I was also shown. The effect was smaller than the combination with three immune status subtypes, but better applicable in daily routine pathology practice. Patients with stroma-low tumors also expressing classical HLA class I have a better prognosis than patients with stroma-high tumors with loss of expression or downregulation of classical HLA class I.

The estimation of TSR is simple, inexpensive and takes only a few minutes. It can be done on regular H&E slides during routine pathology investigation of the resected tissue. Since the introduction of pre-operative chemotherapy, which leads to the formation of non-desmoplastic stroma and, therefore, the resection material unsuitable for TSR scoring, it might be of interest to score the TSR on tumor biopsies. In esophageal adenocarcinoma biopsies, the reproducibility of TSR scoring on biopsies was good [33], and it is plausible that this is even better in breast cancer due to lack of the muscular area [34]. Promising is the current interest in automation of the TSR parameter [13]. Assessment of the six cellular immune markers is relatively time consuming. The assessment of only classical HLA class I takes less effort and may help optimize risk stratification in combination with TSR.

Patients with early stage breast cancer are often treated with adjuvant systemic therapy (endocrine therapy, chemotherapy or agents against HER2) based on tumor characteristics such as HER2 status, tumor size and lymph node status. A substantial number of women with breast cancer is overtreated. These patients do not benefit from adjuvant therapy but are exposed to the risk of toxic effects. The TSR, immune status or a combination of these prognostic markers might be used to select patients who could be spared adjuvant therapy or to select patients more confident to treatment and which can be monitored for recurrences more frequently. Especially patients with stroma-high tumors and low immune status could possibly benefit from more aggressive treatment whereas for patients with stroma-low tumors and high immune status less aggressive treatment could be discussed. The method described in this paper could give valuable additional pathology-based information for patients with invasive breast cancer.

## Conclusion

Simple H&E stained sections contain more information than previously fathomed. The TSR is a simple, fast and cheap method for the identification of patients with more aggressive disease. Tumor immune status profiling is promising for further prognostication and the achievement of tailored treatment for breast cancer patients. The combination of TSR and immune status of tumors is a strong prognosticator, applicable for daily routine use.

## Electronic supplementary material

Below is the link to the electronic supplementary material.

Supplementary material 1 (DOCX 36 kb)

Supplementary material Tumor-stroma ratio. **a** Stroma-low tumor**, b** Stroma-high tumor (TIFF 2489 kb)

Supplementary material Staining results of immune markers. Abbreviation: HLA = human leukocyte antigen (TIFF 6447 kb)

Supplementary material 4 (TIFF 6982 kb)

## References

[1] Anderson WF, Rosenberg PS, Prat A, Perou CM, Sherman ME (2014). How many epidemiological types of breast cancer: two, three, four, or more. Cancer Res.

[2] Paik S, Shak S, Tang G, Kim C, Baker J, Cronin M, Baehner FL, Walker MG, Watson D, Park T, Hiller W, Fisher ER, Wickerham DL, Bryant J, Wolmark N (2004). A multigene assay to predict recurrence of tamoxifen-treated, node-negative breast cancer. New Engl J Med.

[3] Hanahan D, Weinberg RA (2000). The hallmarks of cancer. Cell.

[4] Hanahan D, Weinberg RA (2011). Hallmarks of cancer: the next generation. Cell.

[5] Goldhirsch A, Ingle JN, Gelber RD, Coates AS, Thurlimann B, Senn HJ (2009). Thresholds for therapies: highlights of the St Gallen International Expert Consensus on the primary therapy of early breast cancer 2009. Ann Oncol.

[6] Galea MH, Blamey RW, Elston CE, Ellis IO (1992). The Nottingham Prognostic Index in primary breast-cancer. Breast Cancer Res Treat.

[7] Olivotto IA, Bajdik CD, Ravdin PM, Speers CH, Coldman AJ, Norris BD, Davis GJ, Chia SK, Gelmon KA (2005). Population-based validation of the prognostic model ADJUVANT! for early breast cancer. J Clin Oncol.

[8] Dekker TJA, van de Velde CJH, van Pelt GW, Kroep JR, Julien JP, Smit VTHBM, Tollenaar RAEM, Mesker WE (2013). Prognostic significance of the tumor-stroma ratio: validation study in node-negative premenopausal breast cancer patients from the EORTC perioperative chemotherapy (POP) trial (10854). Breast Cancer Res Treat.

[9] Gujam FJ, Edwards J, Mohammed ZM, Going JJ, McMillan DC (2014). The relationship between the tumour stroma percentage, clinicopathological characteristics and outcome in patients with operable ductal breast cancer. Br J Cancer.

[10] Wang K, Ma W, Wang JB, Yu L, Zhang XM, Wang ZB, Tan BX, Wang NN, Bai B, Yang SS, Liu HQ, Zhu SJ, Cheng YF (2012). Tumor-stroma ratio is an independent predictor for survival in esophageal squamous cell carcinoma. J Thorac Oncol.

[11] Moorman AM, Vink R, Heijmans HJ, van der Palen J, Kouwenhoven EA (2012). The prognostic value of tumour-stroma ratio in triple-negative breast cancer. Ejso.

[12] Mesker WE, Junggeburt JMC, Szuhai K, de Heer P, Morreau H, Tanke HJ, Tollenaar R (2007). The carcinoma-stromal ratio of colon carcinoma is an independent factor for survival compared to lymph node status and tumor stage. Cell Oncol.

[13] West NP, Dattani M, McShane P, Hutchins G, Grabsch J, Mueller W, Treanor D, Quirke P, Grabsch H (2010). The proportion of tumour cells is an independent predictor for survival in colorectal cancer patients. Br J Cancer.

[14] Park JH, Richards CH, McMillan DC, Horgan PG, Roxburgh CS (2014). The relationship between tumour stroma percentage, the tumour microenvironment and survival in patients with primary operable colorectal cancer. Ann Oncol.

[15] Mueller MM, Fusenig NE (2004). Friends or foes—Bipolar effects of the tumour stroma in cancer. Nat Rev Cancer.

[16] Kirilovsky A, Marliot F, El Sissy C, Haicheur N, Galon J, Pages F (2016). Rational bases for the use of the Immunoscore in routine clinical settings as a prognostic and predictive biomarker in cancer patients. Int Immunol.

[17] Pruneri G, Vingiani A, Bagnardi V, Rotmensz N, De Rose A, Palazzo A, Colleoni AM, Goldhirsch A, Viale G (2016). Clinical validity of tumor-infiltrating lymphocytes analysis in patients with triple-negative breast cancer. Ann Oncol.

[18] Loi S, Sirtaine N, Piette F, Salgado R, Viale G, Van Eenoo F, Rouas G, Francis P, Crown JP, Hitre E, de Azambuja E, Quinaux E, Di Leo A, Michiels S, Piccart MJ, Sotiriou C (2013). Prognostic and predictive value of tumor-infiltrating lymphocytes in a phase III randomized adjuvant breast cancer trial in node-positive breast cancer comparing the addition of docetaxel to doxorubicin with doxorubicin-based chemotherapy: BIG 02-98. J Clin Oncol.

[19] de Kruijf FM, Engels CC, van de Water W, Bastiaannet E, Smit VT, van de Velde CJ, Liefers GJ, Kuppen PJ (2013). Tumor immune subtypes distinguish tumor subclasses with clinical implications in breast cancer patients. Breast Cancer Res Treat.

[20] Cirri P, Chiarugi P (2012). Cancer-associated-fibroblasts and tumour cells: a diabolic liaison driving cancer progression. Cancer Metastasis Rev.

[21] Hu M, Polyak K (2008). Microenvironmental regulation of cancer development. Curr Opin Genet Dev.

[22] Kim JB, Stein R, O’Hare MJ (2005). Tumour-stromal interactions in breast cancer: the role of stroma in tumourigenesis. Tumour Biol.

[23] Hynes SO, Coleman HG, Kelly PJ, Irwin S, O’Neill RF, Gray RT, McGready C, Dunne PD, McQuaid S, James JA, Salto-Tellez M, Loughrey MB (2017). Back to the future: routine morphological assessment of the tumour microenvironment is prognostic in stage II/III colon cancer in a large population-based study. Histopathology.

[24] McShane LM, Altman DG, Sauerbrei W, Taube SE, Gion M, Clark GM (2006). REporting recommendations for tumor MARKer prognostic studies (REMARK). Breast Cancer Res Treat.

[25] de Kruijf EM, van Nes JG, van de Velde CJ, Putter H, Smit VT, Liefers GJ, Kuppen PJ, Tollenaar RA, Mesker WE (2011). Tumor-stroma ratio in the primary tumor is a prognostic factor in early breast cancer patients, especially in triple-negative carcinoma patients. Breast Cancer Res Treat.

[26] de Kruijf EM, van Nes JG, Sajet A, Tummers QR, Putter H, Osanto S, Speetjens FM, Smit VT, Liefers GJ, van de Velde CJ, Kuppen PJ (2010). The predictive value of HLA class I tumor cell expression and presence of intratumoral Tregs for chemotherapy in patients with early breast cancer. Clin Cancer Res.

[27] de Kruijf EM, Sajet A, van Nes JG, Natanov R, Putter H, Smit VT, Liefers GJ, van den Elsen PJ, van de Velde CJ, Kuppen PJ (2010). HLA-E and HLA-G expression in classical HLA class I-negative tumors is of prognostic value for clinical outcome of early breast cancer patients. J Immunol.

[28] Algarra I, Garcia-Lora A, Cabrera T, Ruiz-Cabello F, Garrido F (2004). The selection of tumor variants with altered expression of classical and nonclassical MHC class I molecules: implications for tumor immune escape. Cancer Immunol Immunother.

[29] Wischhusen J, Waschbisch A, Wiendl H (2007). Immune-refractory cancers and their little helpers–an extended role for immunetolerogenic MHC molecules HLA-G and HLA-E?. Semin Cancer Biol.

[30] Khong HT, Restifo NP (2002). Natural selection of tumor variants in the generation of “tumor escape” phenotypes. Nat Immunol.

[31] Marin R, Ruiz-Cabello F, Pedrinaci S, Mendez R, Jimenez P, Geraghty DE, Garrido F (2003). Analysis of HLA-E expression in human tumors. Immunogenetics.

[32] Cerwenka A, Baron JL, Lanier LL (2001). Ectopic expression of retinoic acid early inducible-1 gene (RAE-1) permits natural killer cell-mediated rejection of a MHC class I-bearing tumor in vivo. Proc Natl Acad Sci USA.

[33] Courrech Staal EF, Smit VT, van Velthuysen ML, Spitzer-Naaykens JM, Wouters MW, Mesker WE, Tollenaar RA, van Sandick JW (2011). Reproducibility and validation of tumour stroma ratio scoring on oesophageal adenocarcinoma biopsies. Eur J Cancer.

[34] Dekker TJ, Charehbili A, Smit VT, ten Dijke P, Kranenbarg EM, van de Velde CJ, Nortier JW, Tollenaar RA, Mesker WE, Kroep JR (2015). Disorganised stroma determined on pre-treatment breast cancer biopsies is associated with poor response to neoadjuvant chemotherapy: Results from the NEOZOTAC trial. Mol Oncol.

