# Supplementary material for: Prognostic value of tumor–stroma ratio combined with the immune status of tumors in invasive breast carcinoma
Source: Breast Cancer Res Treat. 2017 Dec 22;168(3):601–12. doi: 10.1007/s10549-017-4617-6 (PMC5842256; doi:10.1007/s10549-017-4617-6)
Supplement: Supplementary file 1 — Supplementary material 1 (DOCX 36 kb) [file 10549_2017_4617_MOESM1_ESM.docx]

| **Supplementary table 1a** Prognostic value of the TSR stratified for breast cancer subtypes in total group (*N* = 420) | | | | | | | |
| --- | --- | --- | --- | --- | --- | --- | --- |
|  |  | Recurrence Free Period | | | Overall Survival | | |
|  | *N* | HR | 95% CI | *P*-value | HR | 95% CI | *P*-value |
| **Luminal A** |  |  |  |  |  |  |  |
| Stroma-low | 164 | 1 |  | **.008** | 1 |  | .139 |
| Stroma-high | 144 | 1.57 | 1.13-2.19 |  | 1.24 | 0.93-1.65 |  |
| **Luminal B** |  |  |  |  |  |  |  |
| Stroma-low | 10 | 1 |  | .394 | 1 |  | .242 |
| Stroma-high | 5 | 1.78 | 0.47-6.71 |  | 2.05 | 0.62-6.82 |  |
| **HER2-like** |  |  |  |  |  |  |  |
| Stroma-low | 14 | 1 |  | .396 | 1 |  | .183 |
| Stroma-high | 21 | 1.57 | 0.55-4.46 |  | 1.77 | 0.77-4.09 |  |
| **Triple-negative** |  |  |  |  |  |  |  |
| Stroma-low | 36 | 1 |  | **.004** | 1 |  | .231 |
| Stroma-high | 26 | 2.41 | 1.32-4.40 |  | 1.46 | 0.78-2.73 |  |

| **Supplementary table 1b** Prognostic value of the TSR stratified for breast cancer subtypes within known immune status group (*N* = 279) | | | | | | | |
| --- | --- | --- | --- | --- | --- | --- | --- |
|  |  | Recurrence Free Period | | | Overall Survival | | |
|  | *N* | HR | 95% CI | *P*-value | HR | 95% CI | *P*-value |
| **Luminal A** |  |  |  |  |  |  |  |
| Stroma-low | 99 | 1 |  | .100 | 1 |  | .508 |
| Stroma-high | 93 | 1.42 | 0.94-2.15 |  | 1.13 | 0.79-1.62 |  |
| **Luminal B** |  |  |  |  |  |  |  |
| Stroma-low | 6 | 1 |  | .133 | 1 |  | .111 |
| Stroma-high | 4 | 5.69 | 0.59-55.19 |  | 4.07 | 0.73-22.88 |  |
| **HER2-like** |  |  |  |  |  |  |  |
| Stroma-low | 5 | 1 |  | .953 | 1 |  | .891 |
| Stroma-high | 10 | 1.05 | 0.20-5.45 |  | 1.10 | 0.29-4.20 |  |
| **Triple-negative** |  |  |  |  |  |  |  |
| Stroma-low | 36 | 1 |  | **.010** | 1 |  | .231 |
| Stroma-high | 26 | 2.60 | 1.26-5.39 |  | 1.46 | 0.78-2.73 |  |

Abbreviations: TSR = tumor-stroma ratio, HER2 = human epidermal growth factor receptor 2

| **Supplementary table 2** Prognostic value of the immune status stratified for breast cancer subtypes (*N* = 279) | | | | | | | |
| --- | --- | --- | --- | --- | --- | --- | --- |
|  |  | Recurrence Free Period | | | Overall Survival | | |
|  | *N* | HR | 95% CI | *P*-value | HR | 95% CI | *P*-value |
| **Luminal A** |  |  |  |  |  |  |  |
| High IS | 33 | 1 |  | **<.001** | 1 |  | **.010** |
| Intermediate IS | 123 | 1.53 | 0.80-2.94 |  | 1.72 | 0.97-3.05 |  |
| Low IS | 36 | 3.53 | 1.75-7.14 |  | 2.65 | 1.40-5.02 |  |
| **Luminal B** |  |  |  |  |  |  |  |
| High IS | 3 | 1 |  | .651 | 1 |  | 0.979 |
| Intermediate IS | 7 | 1.69 | 0.17-16.40 |  | 0.98 | 0.18-5.44 |  |
| Low IS | 0 |  |  |  |  |  |  |
| **HER2-like** |  |  |  |  |  |  |  |
| High IS | 2 |  |  | .999 | 1 |  | .801 |
| Intermediate IS | 11 |  |  |  | 0.75 | 0.15-3.84 |  |
| Low IS | 2 |  |  |  | 1.27 | 0.17-9.33 |  |
| **Triple-negative** |  |  |  |  |  |  |  |
| High IS | 12 | 1 |  | **.003** | 1 |  | **.011** |
| Intermediate IS | 36 | 2.25 | 0.65-7.78 |  | 3.07 | 1.06-8.87 |  |
| Low IS | 14 | 6.39 | 1.79-22.84 |  | 5.54 | 1.79-17.18 |  |

Abbreviations: IS = immune status, HER2 = human epidermal growth factor receptor 2
