# Supplementary figures and images for: Prognostic value of tumor–stroma ratio combined with the immune status of tumors in invasive breast carcinoma
Source: Breast Cancer Res Treat. 2017 Dec 22;168(3):601–12. doi: 10.1007/s10549-017-4617-6 (PMC5842256; doi:10.1007/s10549-017-4617-6)

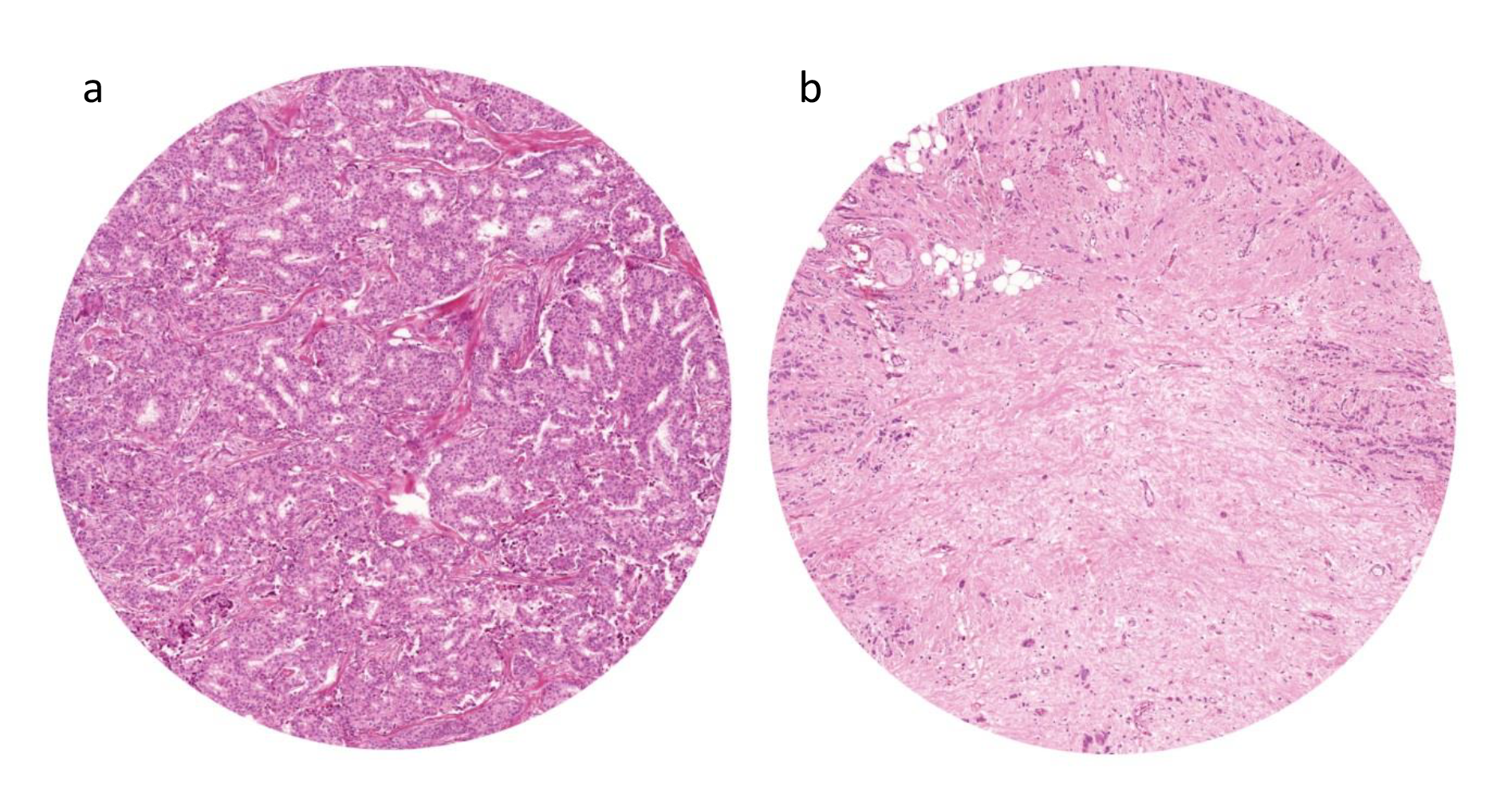

Supplement: Supplementary file 2 — Supplementary material Tumor-stroma ratio. a Stroma-low tumor, b Stroma-high tumor (TIFF 2489 kb) [file 10549_2017_4617_MOESM2_ESM.tif]

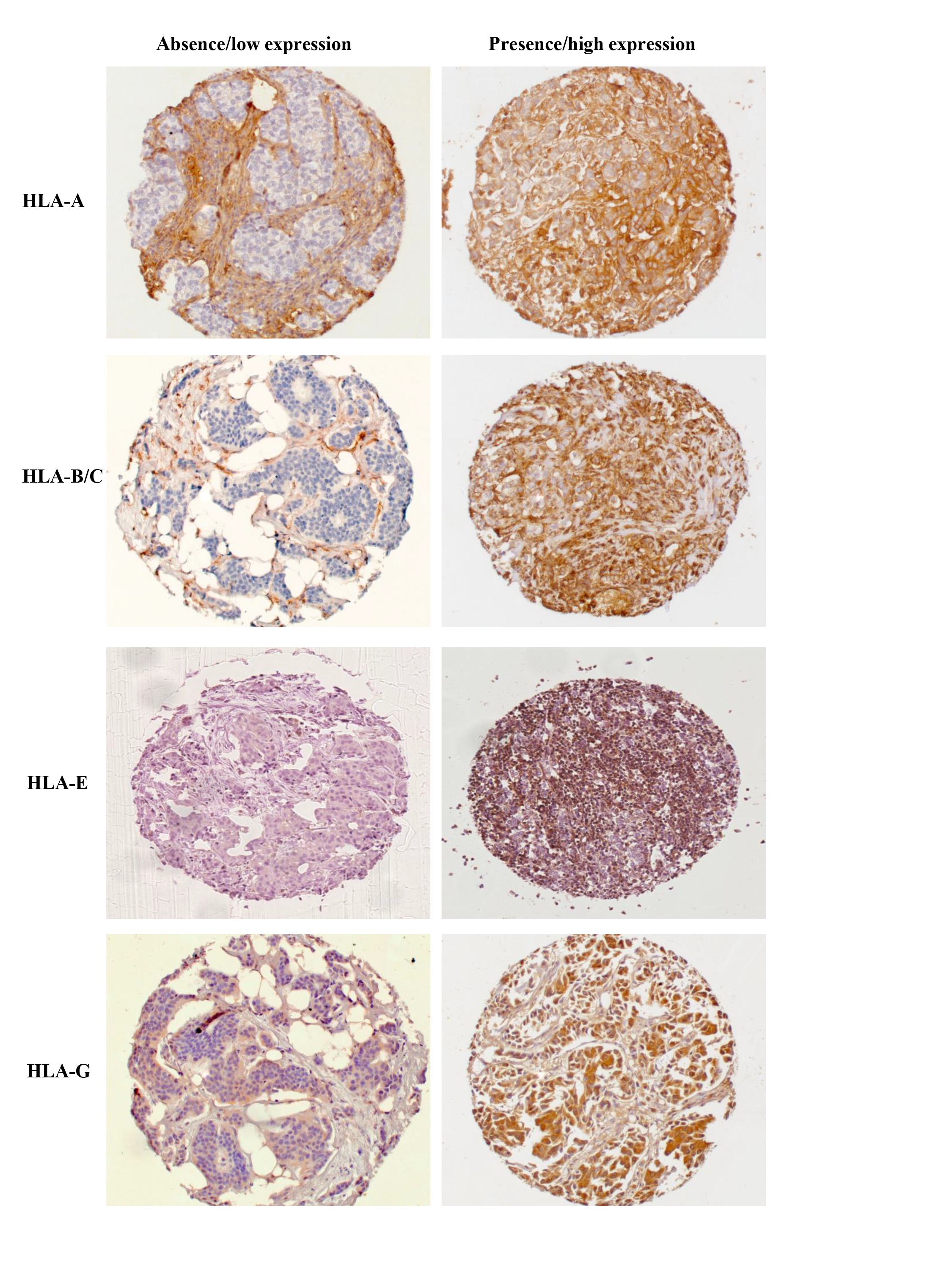

Supplement: Supplementary file 3 — Supplementary material Staining results of immune markers. Abbreviation: HLA = human leukocyte antigen (TIFF 6447 kb) [file 10549_2017_4617_MOESM3_ESM.tif]

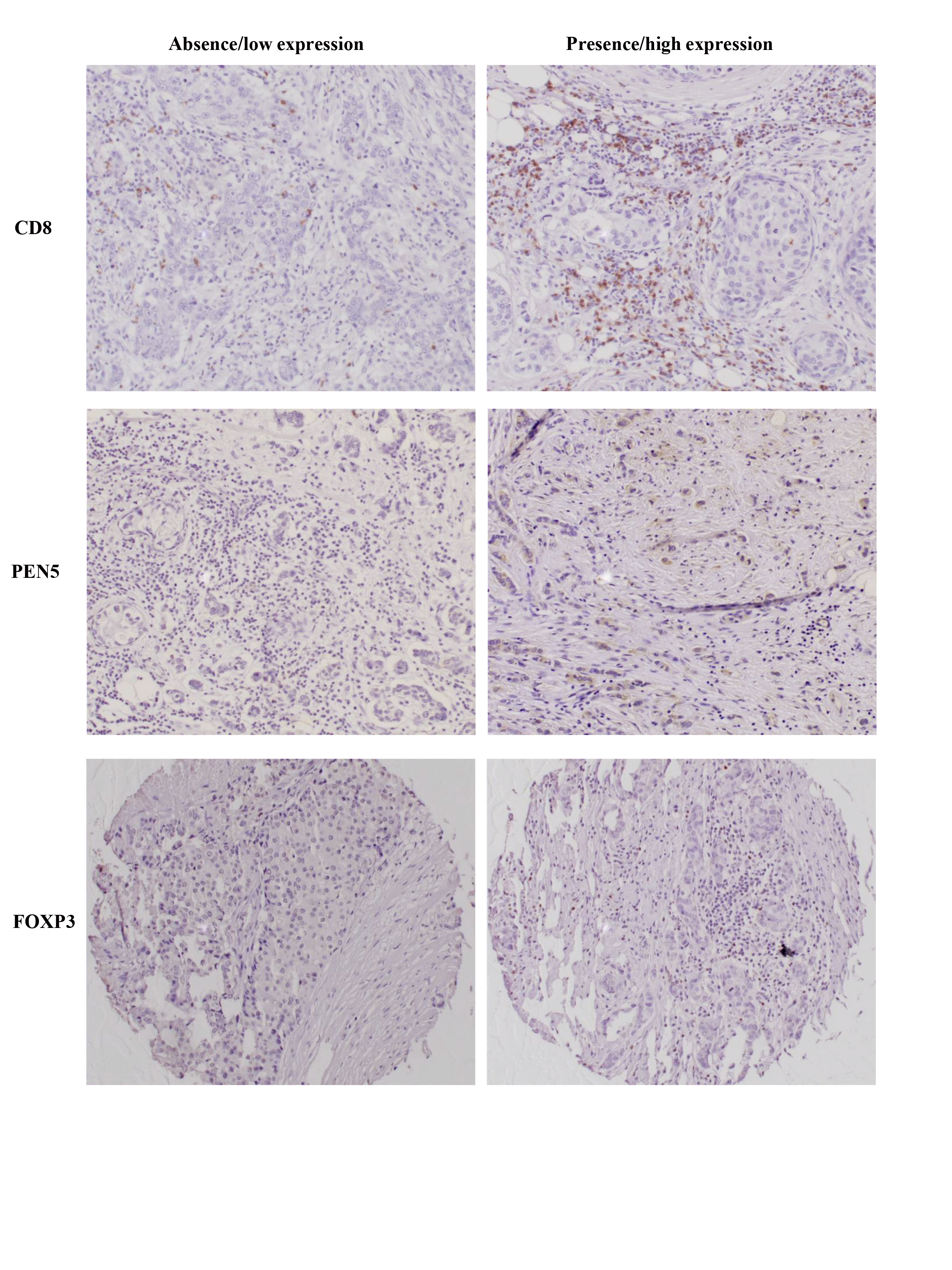

Supplement: Supplementary file 4 — Supplementary material 4 (TIFF 6982 kb) [file 10549_2017_4617_MOESM4_ESM.tif]
